# Respiratory and peripheral muscle ultrasound, including shear-wave elastography, for sarcopenia screening in COPD

**DOI:** 10.3389/fnut.2025.1687103

**Published:** 2025-12-05

**Authors:** Zeyang Dong, Jie Chen, Jin Ge, Mengyao Zhao, Yuke Zhao, Sihui Zheng, Jian Ye, Xixi Sun, Bin Huang

**Affiliations:** 1 The Second School Of Clinical Medicine, Zhejiang Chinese Medical University, Hangzhou, China; 2 Department of Ultrasound, Zhejiang Hospital, Hangzhou, China; 3 Department of Respiratory Medicine, Zhejiang Hospital, Hangzhou, China

**Keywords:** COPD, sarcopenia, nutritional assessment, muscle ultrasound, respiratory muscle

## Abstract

**Background:**

Chronic obstructive pulmonary disease (COPD) is associated with an increased risk of sarcopenia, which contributes to disease progression and poor clinical outcomes. Ultrasound offers a noninvasive and effective way to assess muscle structure and function. This study aimed to evaluate the diagnostic utility of two-dimensional ultrasound (2D-US) and shear wave elastography (SWE) for detecting sarcopenia in COPD patients.

**Methods:**

In this prospective, single-center study, 76 COPD patients were enrolled and classified into sarcopenia and non-sarcopenia groups based on diagnostic criteria. All participants underwent 2D-US to assess the thickness of the diaphragm (DTei), intercostal muscles (ICMTei), rectus abdominis (RAT), rectus femoris (RFT), and biceps brachii (BBT); as well as the cross-sectional area of the rectus femoris (RF-CSA), and biceps brachii (BB-CSA). Additionally, respiratory muscle function parameters, including diaphragm thickening fraction (DTF), diaphragm excursion (DE), and intercostal muscle thickening fraction (ICMTF), were also measured. Where feasible, SWE was performed on 42 patients to measure the shear wave velocity (SWV) of each muscle. Multivariable logistic regression identified independent predictors of sarcopenia, and diagnostic performance was evaluated using ROC curve analysis.

**Results:**

Between April and July 2025, 76 COPD patients were enrolled in the study assess ultrasound screening sarcopenia in COPD patients, and 30 healthy subjects were recruited in the reproducibility study. In the repeatability assessment, the ICC values for all parameters ranged from 0.851 to 0.994. In the sarcopenia screening study, significant differences were observed between groups for multiple parameters, including DTei, DE, RFT, RF-CSA, BB-CSA, D-SWV, ICM-SWV, RA-SWV, and BB-SWV (all *p* < 0.05). Logistic regression identified ICMTF (OR = 6.738), BBT (OR = 6.231), DTF (OR = 3.505), DE (OR = 0.312), RF-CSA (OR = 0.127), and BB-CSA (OR = 0.009) as independent predictors of sarcopenia (AUC = 0.956). After including SWE parameters, RA-SWV (OR = 19.171), BB-SWV (OR = 4.837), RF-CSA (OR = 0.263), DTei (OR = 0.197), and ICM-SWV (OR = 0.165) were identified as additional predictors, improving diagnostic accuracy (AUC = 0.961).

**Conclusion:**

Combining morphological and elasticity-based ultrasound parameters provides a reliable, non-invasive method for diagnosing sarcopenia in COPD patients. This approach may help guide early interventions and personalized management strategies.

## Introduction

Chronic obstructive pulmonary disease (COPD) is a prevalent condition characterized by persistent airway inflammation and several extrapulmonary manifestations that adversely affect patients’ prognosis. Notably, COPD frequently leads to malnutrition, altered body composition, reduced physical activity, and a decline in muscle mass or strength—features closely associated with sarcopenia, a progressive skeletal muscle disorder that significantly affects both quality of life and clinical outcomes (1, 2). Sarcopenia is estimated to affect 5–13% of the healthy elderly population, but its prevalence is considerably higher in individuals with COPD, with studies showing rates between 8.38 and 52.1% (3–5). Sarcopenia in COPD patients is linked to a decline in physical function, hospitalization, and increased mortality, making it an important target for early detection and intervention (6, 7).

Nutrition plays a pivotal role in muscle health, and malnutrition is known to accelerate the progression of sarcopenia in COPD patients (8). However, assessing sarcopenia in COPD patients remains challenging, with inconsistent definitions and diagnostic methods across studies and clinical settings. The European Working Group on Sarcopenia in Older People (EWGSOP) and the Asian Working Group for Sarcopenia (AWGS) have proposed criteria to diagnose sarcopenia based on muscle mass, strength, and performance, but the implementation of these criteria is hindered by the limitations of traditional assessment techniques (9, 10). Among these, grip strength testing is widely recognized as a quick and reliable method for evaluating muscle strength in clinical settings. Among these, grip strength testing is widely recognized as a quick and reliable method for evaluating muscle strength in clinical settings. Computed tomography (CT) is currently considered the gold standard for quantifying muscle mass. However, there are several drawbacks in clinical practice, including radiation exposure, poor reproducibility, and prolonged patient transport time, making them inconvenient as diagnostic tools in clinical settings. Bioelectrical impedance analysis (BIA) instruments can be used for bedside examinations, but they only measure fat-free mass through bioelectrical impedance analysis, rather than directly measuring muscle mass, which may result in some errors (11).

In this context, with the development of muscle ultrasound technology, it has gained significant attention as a tool for assessing muscle mass due to its safety, non-invasiveness, real-time imaging capabilities, and cost-effectiveness. The use of muscle ultrasound technology for assessing, preventing, and monitoring muscle mass in patients with sarcopenia holds great clinical value and huge potential (12).

Muscle thickness (MT) and cross-sectional area (CSA) are two traditional two-dimensional ultrasound (2D-US) parameters for assessing muscle mass and have been widely used in sarcopenia research (13). In COPD patients, most studies have primarily focused on the analysis of lower limb muscles, particularly the quadriceps, with these measurements often used for sarcopenia screening (14). In addition, ultrasound assessment of the rectus femoris and rectus abdominis is also frequently employed in the diagnosis of sarcopenia (15). However, a statement from the American Thoracic Society (ATS) emphasized that in addition to reduced lower limb muscle mass and function, COPD patients also exhibit changes in respiratory muscle mass and function (16). Studies have shown that diaphragm thickness, as measured by ultrasound, is significantly reduced in COPD patients with sarcopenia compared to healthy elderly individuals (17), and that intercostal muscle thickness is positively correlated with forced expiratory volume in one second (FEV₁) (18). Despite these findings, relatively few studies have explored the use of respiratory muscle ultrasound for screening sarcopenia in COPD patients.

Shear wave elastography (SWE) is an emerging ultrasound-based technique that enables the quantitative assessment of soft tissue physiology and pathology. By measuring the shear wave velocity (SWV) as they propagate through tissue—expressed in meters per second (m/s)—SWE provides an objective measure of tissue stiffness (19).

This study aims to evaluate 2D-US and SWE parameters of the diaphragm, intercostal muscles, rectus abdominis, biceps brachii, and rectus femoris in patients with COPD. It further seeks to investigate the correlations between these ultrasound parameters and the presence of sarcopenia. Additionally, the study aims to compare the ultrasound characteristics between COPD patients with and without sarcopenia and to assess the feasibility of using ultrasound as a screening tool for sarcopenia in this population.

## Materials and methods

### Study participants and design

**Figure 1:**
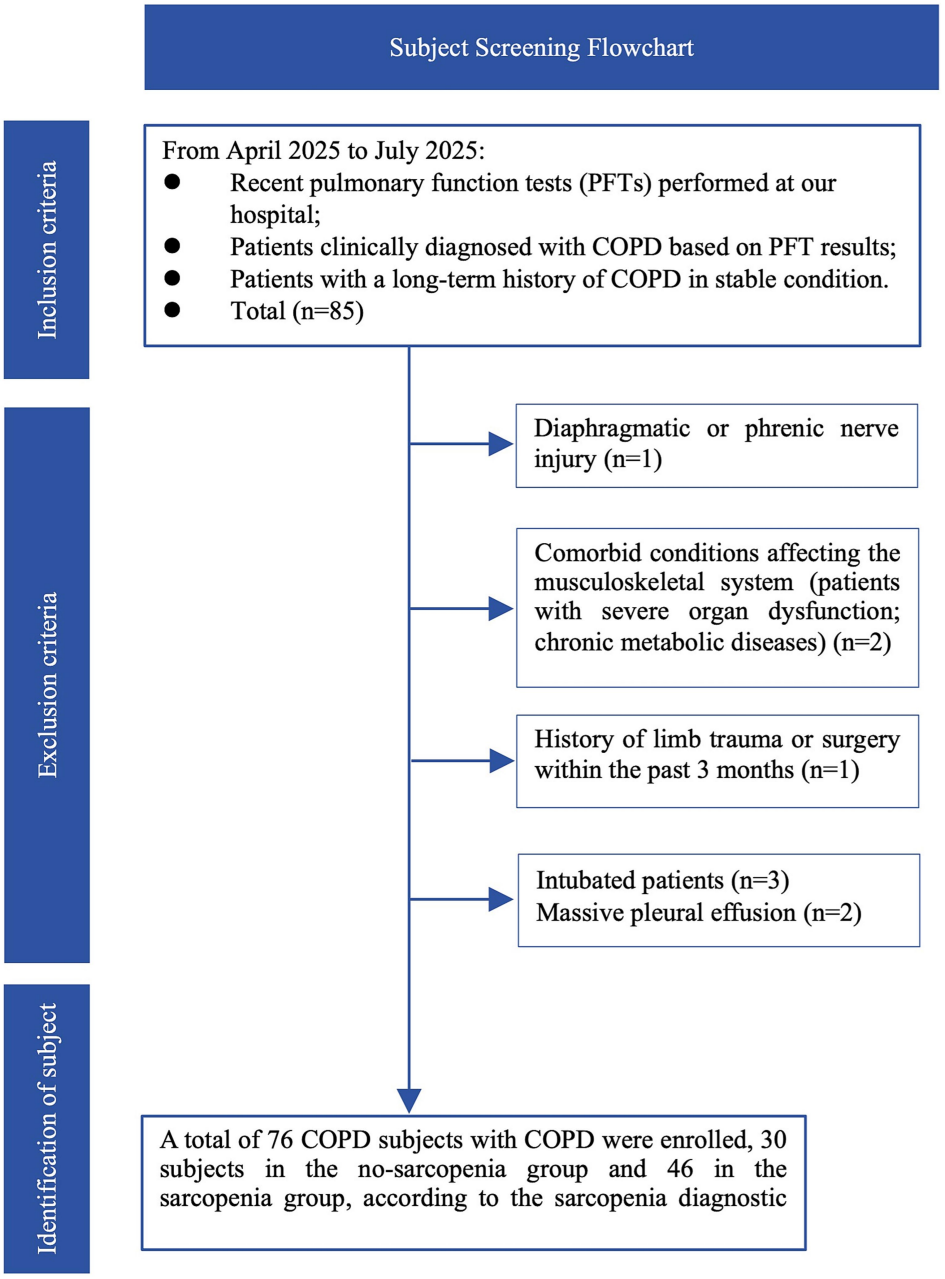
Subject screening flowchart.

We conducted a single-center, prospective cross-sectional study at Zhejiang Hospital in China from April to July 2025. The study was approved by the Ethics Review Committee of Zhejiang Hospital, and written informed consent was obtained from all participants (Approval No. ZJHIRB-2025-060K, 2025-071K). The inclusion and exclusion criteria are illustrated in the flowchart (Figure 1). All participants underwent respiratory muscles and peripheral muscles ultrasonography to assess the structural, functional, and elastic properties of the muscles. One of the reproducibility assessments of muscle ultrasound was performed in normal subjects to ensure the reliability of respiratory muscle ultrasonography results.

### Study participants recruitment

All subjects were recruited at Zhejiang Hospital on a voluntary basis. In the reproducibility study, a total of 30 healthy subjects were recruited. In the study assess ultrasound screening sarcopenia in COPD patients, a total of 76 COPD patients were enrolled.

Inclusion criteria for healthy individual included (1) healthy individuals aged 18–60 years, regardless of gender; and (2) no history of respiratory infections or newly diagnosed cardiac diseases within the past month. The exclusion criteria included: (1) chronic respiratory or cardiovascular diseases; (2) a history of smoking; (3) current use of respiratory or neurological medications; (4) diaphragm or phrenic nerve injury; (5) severe myasthenia gravis, stroke, or other conditions associated with respiratory center depression; (6) a history of chest wall surgery or trauma; (7) inability to breathe normally or cooperate during examinations; and (8) inability to complete two full sessions of respiratory muscle ultrasound assessments.

Inclusion criteria for COPD patients included (1) stable patients with a long history of COPD; (2) COPD patients who underwent pulmonary function tests in our hospital in the last 3 months. Exclusion criteria included (1) diaphragm or phrenic nerve injury; (2) those with lung malignancy; (3) history of chest surgery or trauma; (4) severe myasthenia gravis, stroke, or other disorders associated with respiratory central depression; (5) restrictive lung disease; (6) those with severe cardiac insufficiency; and (7) those who were unable to complete two complete ultrasound evaluations of the respiratory muscles.

### Ultrasound assessment of muscles

**Figure 2:**
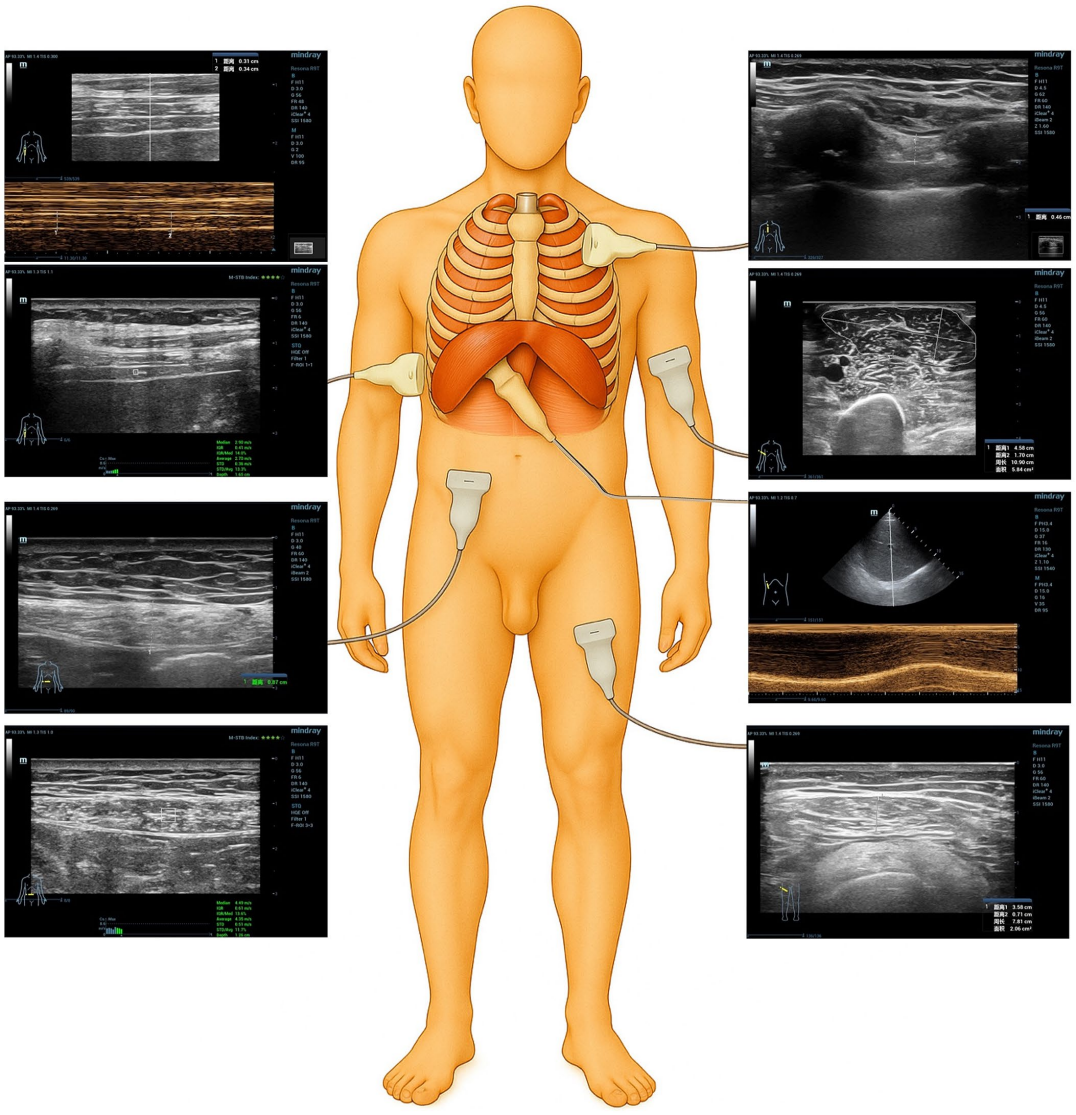
Schematic diagram of ultrasonography of the diaphragm and intercostal muscles.

Ultrasound examinations were performed either at the bedside in the respiratory medicine ward or in the ultrasound clinic. Prior to the assessment, all participants underwent respiratory coordination training. Ultrasound equipment included the Mindray Resona R9T (Mindray Bio-Medical Electronics, Shenzhen, China) and the portable Mindray M9 systems (Mindray Bio-Medical Electronics, Shenzhen, China). All measurements of 2D-SWE were obtained using the Resona R9T system. Due to limited mobility and prolonged bed rest in some patients with COPD, and owing to the absence of a SWE imaging module in the portable Mindray M9 device at this research center, SWE data could not be acquired for this group of subjects. The visualization procedure diagram for the ultrasound examination is shown in Figure 2.

#### Diaphragm ultrasound examination method

The patient was positioned supine, and a high-frequency linear transducer (5–18 MHz) was placed perpendicular to the ribs at the 8th to 10th intercostal space along the right anterior or mid-axillary line. In B-mode, diaphragm thickness (DT) was measured between two hyperechoic lines—the pleura and peritoneum.

M-mode was used to assess diaphragm movement over three respiratory cycles, measuring diaphragm thickness at end-inspiration (DTei) and end-expiration (DTee), with diaphragm thickening fraction (DTF) calculated as:


DTF=(DTei–DTee)/DTee×100%

At the same location, SWE was performed after clear diaphragm visualization. A 1 × 1 mm sampling box was placed at the diaphragm center. The patient was instructed to breathe quietly and hold their breath at the end of expiration, and the diaphragm shear wave velocity (D-SWV) was automatically calculated after 3–5 measurements.

For diaphragm excursion (DE), the probe was placed at the intersection of the right midclavicular line and costal margin, using the liver as an acoustic window. M-mode sampling line was positioned perpendicular to the diaphragmatic surface at the lower edge of the liver. DE was calculated as the vertical distance from baseline to the peak of the curve during respiration.

#### Intercostal muscle ultrasound examination method

The patient was positioned supine, and the probe was placed on the right chest wall at the 2nd to 3rd intercostal space, 2–4 cm lateral to the sternum. After obtaining a cross-sectional view of the intercostal muscles in B-mode, M-mode was used to measure intercostal muscle thickness (ICMT) at end-inspiration (ICMTei) and end-expiration (ICMTee). The intercostal muscle thickening fraction (ICMTF) was then calculated as:


ICMTF=(ICMTei–ICMTee)/ICMTee×100%

After visualizing the muscle structure clearly over 2–3 respiratory cycles, the SWE was performed, with a 1 × 1 mm sampling frame placed at the center of the intercostal muscle. After 3–5 measurements, the system automatically calculated the intercostal muscle shear wave velocity (ICM-SWV).

#### Rectus abdominis ultrasound examination method

The patient was positioned supine with the muscles relaxed. The probe was placed horizontally to the right of the umbilicus, perpendicular to the muscle fibers, to obtain a transverse section image. In this plane, the rectus abdominis thickness (RAT) was measured.

Then, the SWE was performed, with a 2 × 2 mm sampling frame was placed at the center of the rectus abdominis bundle, 1–3 cm below the skin. After 3–5 repetitions, the system automatically calculated the rectus abdominis shear wave velocity (RA-SWV).

#### Rectus femoris ultrasound examination method

The patient was positioned supine with the lower limbs straightened and relaxed. The probe was placed at the midpoint of the line connecting the anterior superior iliac spine (ASIS) and the upper edge of the patella, perpendicular to the muscle fibers of the rectus femoris, to obtain a transverse section image. In this plane, the rectus femoris thickness (RFT) and cross-sectional area (RF-CSA) were measured.

Next, the SWE was performed, with a 2 × 2 mm sampling frame was positioned at the center of the rectus femoris bundle, 1–3 cm below the skin. After 3–5 repetitions, the system automatically calculated the rectus femoris shear wave velocity (RF-SWV).

#### Biceps brachii ultrasound examination method

The patient was positioned supine with the upper limbs relaxed and the palms facing upward. The ultrasound probe was placed at the midpoint of the upper arm, oriented perpendicular to the muscle fibers to capture a cross-sectional image of the biceps brachii. On this cross-section, the biceps brachii thickness (BBT) and cross-sectional area (BB-CSA) were measured.

Then, the SWE was performed, with a 2 × 2 mm sampling frame positioned at the center of the biceps brachii bundle, approximately 1–3 cm from the skin. After 3–5 repetitions, the system automatically calculated the biceps brachii shear wave velocity (BB-SWV).

### Repeatability assessment

Given the instability and heterogeneity of disease progression, coupled with the universal applicability of repeatability assessments, we opted to evaluate the repeatability of ultrasound examinations in healthy subjects. Two sonographers utilized Mindray Resona R9T ultrasound machine to perform examinations on the respiratory muscles and peripheral skeletal muscles of the same healthy subject, adhering to the aforementioned standardized scanning protocol. All subjects were positioned supine, with no more than half an hour elapsing between examinations. Each examination was performed independently by one physician, and neither physician was aware of the other’s ultrasound measurements. Following completion of examinations for all subjects, the two sets of ultrasound results were collated and the reproducibility was evaluated using the intra-class correlation coefficient (ICC).

### Diagnosis of sarcopenia in COPD patients

The diagnosis of sarcopenia was performed according to the revised consensus guidelines by the Asian Working Group for Sarcopenia (AWGS 2019) (11) and the European Working Group on Sarcopenia in Older People (EWGSOP2) (9).

In this study, the assessment was divided into three parts: muscle strength, muscle mass, and physical function. When low muscle mass is combined with low muscle strength and/or low physical function, it can be diagnosed as sarcopenia.

#### Muscle strength assessment

Muscle strength is evaluated using a grip strength test, which is conducted with an electronic grip strength meter (SENSSUN Group Limited, Guangzhou, China, model: EH101). The subject is seated with their elbows bent at 90°, and the dominant hand is used to perform three trials. The highest grip strength value (in kg) is recorded. A grip strength of less than 28 kg for men and less than 18 kg for women is considered indicative of low muscle strength.

#### Muscle mass assessment

Given the practical constraints within clinical settings, many patients possess limited mobility, rendering advanced imaging techniques such as DXA, CT, and MRI difficult to implement. Although these methods are regarded as the gold standard for diagnosing sarcopenia, their high cost, operational complexity, and time-consuming nature present both economic barriers and practical challenges in routine clinical practice. Consequently, this study employs bedside bioelectrical impedance analysis (BIA) for muscle mass assessment, rather than relying on DXA, CT, or MRI.

When using the BIA device (Niujian Biotechnology, Guangzhou, China, model: NUTRILAB). The BIA protocol involved inputting the patient’s height and weight, followed by electrode placement on the wrist, back of the hand, and the same-side ankle and foot for the measurement. An appendicular skeletal muscle index (ASMI) of <7.0 kg/m^2^ for men or <5.7 kg/m^2^ for women is diagnosed as low muscle mass.

#### Physical function assessment

Physical function is primarily assessed using the 4-meter standard walking speed test or the Short Physical Performance Battery (SPPB).

In the 4-meter walking speed test, speed is manually timed with a stopwatch, and a cutoff speed of ≤0.8 m/s is used as the diagnostic threshold. The SPPB combines scores from balance, gait, and the chair rise test, with a score of less than 9 points, based on the AWGS 2019 criteria, serving as the diagnostic threshold. For patients who are bedridden and unable to leave bed, physical functional impairment can be directly diagnosed.

### Assessment of survival status of COPD patients (BODE score)

In order to systematically assess the systemic condition and survival status of COPD patients, we used the BODE multidimensional grading system for score grading (20), consisting of body mass index (B), airflow obstruction (O), dyspnea score (D), and exercise capacity (E), which was categorized into four grades based on the final cumulative scores, with grades 1 ranging from 0 to 2, grades 2 ranging from 3 to 4, grades 3 ranging from 5 to 6, and grades 4 ranging from 7 to 10.

### Statistical analyses

All statistical analyses were performed using SPSS version 26.0 (SPSS, Chicago, IL) and R version 4.4.2 (R Foundation for Statistical Computing, Vienna, Austria). *p*-values less than 0.05 were considered to indicate statistically significant differences. Normally distributed data were presented as mean ± standard deviation ( $\bar{\chi }\pm s$ ), with comparisons between groups made using the independent samples t-test. Skewed data were expressed as median with interquartile range (M [P25, P75]), and group comparisons were performed using the Mann–Whitney *U* test. Differences were considered statistically significant at *p* < 0.05. The correlation between two ordinal variables was assessed using Spearman’s rank correlation coefficient (*ρ*). Intraclass correlation coefficients (ICCs) values were used in repeatable assessments to estimate the agreement between two ultrasound measurements. The following definitions were used to interpret the ICC values for absolute agreement: 0–0.5 as poor; 0.5–0.75 as fair; 0.75–0.95 as good; and 0.95–1.0 as excellent (21). Univariate and multivariate logistic regression analyses were employed to identify significant determinants of the diagnosis of COPD with sarcopenia. Prior to logistic regression analysis, all data were standardized in SPSS 26.0 to eliminate the effects of magnitude differences across variables. Multifactor logistic regression was conducted using the stepwise regression method in R-4.4.2 to construct predictive models. The diagnostic performance of respiratory muscle and skeletal muscle ultrasound parameters for determining sarcopenia group was evaluated using receiver operating characteristic (ROC) curve analysis. Cutoff values were determined to maximize the Youden index. For each threshold, the corresponding sensitivity, specificity, positive predictive value (PPV), negative predictive value (NPV), and accuracy were calculated.

## Results

### Baseline characteristics

Between June 2024 and June 2025, a total of 76 COPD subjects were enrolled at Zhejiang Hospital based on the inclusion and exclusion criteria. The mean age of the participants was 72.51 ± 8.45 years, the mean BMI was 21.02 ± 3.16 kg/m^2^, the mean handgrip strength was 22.66 ± 9.13 kg, and the mean BODE score was 6.00 (4.00, 7.00).

According to the sarcopenia diagnostic criteria, 30 subjects were classified into the no-sarcopenia group and 46 into the sarcopenia group. The mean handgrip strength was 30.88 ± 6.56 kg in the no-sarcopenia group and 17.29 ± 6.06 kg in the sarcopenia group. Statistically significant differences were observed between the two groups in age and handgrip strength. Additionally, significant differences were found between the two groups in mMRC scores, BODE scores, 6MWT scores, and FEV1 scores, as detailed in Table 1.

**Table 1:** Baseline clinical profile of COPD patients.

Variables	All *n* = 76	No-sarcopenia group (*n* = 30)	Sarcopenia group (*n* = 46)	Statistic	*p* value
Genders, *n*(%)				χ^2^ = 0.22	0.638
Male	23 (30.26)	10 (33.33)	13 (28.26)		
Female	53 (69.74)	20 (66.67)	33 (71.74)		
Age (y)	72.51 ± 8.45	66.80 ± 7.04	76.24 ± 7.14	*t* = −5.66	<0.001
Handgrip strength (kg)	22.66 ± 9.13	30.88 ± 6.56	17.29 ± 6.06	*t* = 9.25	<0.001
BMI (kg/m^2^)	21.02 ± 3.16	21.37 ± 2.85	20.80 ± 3.36	*t* = 0.76	0.449
BMI score	1.00 (0.00, 1.00)	0.00 (0.00, 1.00)	1.00 (0.00, 1.00)	*Z* = –0.83	0.407
mMRC score	2.00 (1.00, 2.00)	1.00 (1.00, 2.00)	2.00 (1.00, 2.00)	*Z* = –2.34	0.019
6MWT score	2.00 (0.75, 3.00)	0.00 (0.00, 1.00)	2.00 (2.00, 3.00)	*Z* = –5.08	<0.001
FEV1 score	2.00 (2.00, 3.00)	2.00 (1.25, 2.00)	2.00 (2.00, 3.00)	*Z* = –2.34	0.019
BODE score	6.00 (4.00, 7.00)	4.00 (3.00, 6.00)	7.00 (6.00, 8.00)	*Z* = –4.51	<0.001
BODE grade	3.00 (2.00, 4.00)	2.00 (2.00, 3.00)	4.00 (3.00, 4.00)	*Z* = –4.13	<0.001

COPD, chronic obstructive pulmonary disease; BMI, body mass index; mMRC, modified Medical Research Council; 6MWD, 6-min walk distance; FEV1, forced expiratory volume in 1 s; BODE, Body-Mass Index, airflow Obstruction, Dyspnea, and Exercise Capacity Index. Values are presented as means ± standard deviations or median (inter-quartile).

Due to mobility restrictions in some patients, SWE measurements could not be obtained using bedside ultrasound devices for this cohort. To ensure comparability between the two groups, we performed a comparative analysis of baseline characteristics between the SWE and non-SWE groups. The results showed significant differences between the groups only in the prevalence of sarcopenia and the ICMTF index (*p* < 0.05). No statistically significant differences were observed in any other parameters (Table 2).

**Table 2:** Comparison of baseline clinical characteristics between SWE and non-SWE groups.

Variables	Non-SWE group (*n* = 34)	SWE group (*n* = 42)	Statistic	*P*
Genders, *n* (%)			χ^2^ = 0.02	0.884
Male	24 (70.59)	29 (69.05)		
Female	10 (29.41)	13 (30.95)		
Age (y)	72.21 ± 6.55	72.76 ± 9.79	*t* = −0.30	0.769
BMI (kg/m^2^)	20.50 ± 2.92	21.45 ± 3.32	*t* = −1.31	0.196
Handgrip strength (kg)	21.68 ± 7.12	23.29 ± 10.58	*t* = −0.78	0.436
Sarcopenia, *n* (%)			χ^2^ = 4.35	0.037
Yes	9 (26.47)	21 (50.00)		
No	25 (73.53)	21 (50.00)		
DTei (cm)	0.20 ± 0.06	0.21 ± 0.06	*t* = −0.48	0.630
DTF (%)	0.17 (0.14, 0.27)	0.19 (0.13, 0.28)	*Z* = –0.65	0.514
DE (cm)	1.35 ± 0.58	1.61 ± 0.59	*t* = −1.92	0.059
ICMT (cm)	0.33 ± 0.11	0.36 ± 0.10	*t* = −1.36	0.179
ICMTF (%)	0.16 (0.09, 0.21)	0.11 (0.07, 0.16)	*Z* = –2.08	0.038
RAT (cm)	0.82 ± 0.19	0.77 ± 0.22	*t* = 0.98	0.328
RFT (cm)	0.81 (0.62, 1.05)	0.77 (0.60, 0.97)	*Z* = –0.91	0.361
RF-CSA (cm^2^)	2.11 (1.67, 2.93)	1.97 (1.39, 2.93)	*Z* = –0.49	0.623
BBT (cm)	1.39 (1.21, 1.69)	1.56 (1.25, 1.77)	*Z* = –0.82	0.414
BB-CSA (cm^2^)	5.21 (4.69, 6.44)	4.86 (3.78, 5.78)	*Z* = –1.18	0.236

BMI, body mass index; DTei, diaphragm thickness of end-inspiratory; DTF, diaphragm thickening fraction; DE, diaphragm excursion; ICMT, intercostal muscle thickness; ICMTF, intercostal muscle thickening fraction; RAT, rectus abdominis thickness; RFT, rectus femoris thickness; RF-CSA, rectus femoris cross-sectional area; BBT, biceps brachii thickness; BB-CSA, biceps brachii cross-sectional area.

### Repeatability assessment results

We recruited 30 healthy subjects to assess the reliability of ultrasound findings. By comparing the two sets of measurements for all healthy subjects, the ICCs of DTei, DTF, DE, D-SWV, ICMT, ICMTF, ICM-SWV, RAT, RA-SWV, RFT, RF-CSA, RF-SWV, BBT, BB-CSA, and BB-SWV were 0.953, 0.893, 0.877, 0.897, 0.969, 0.851, 0.912, 0.994, 0.954, 0.986, 0.931, 0.962, 0.991, 0.929, and 0.958, respectively. The results indicate that all examination parameters for respiratory muscles and peripheral skeletal muscles demonstrated excellent reproducibility and high reliability.

### Correlation between sarcopenia and muscle ultrasound parameters in COPD patients

2D-US parameters were analyzed for all patients, intergroup analysis using the rank-sum test combined with Spearman’s correlation coefficient revealed that DTei (*p* < 0.001, *r* = − 0.406), DE (*p* = 0.018, *r* = − 0.269), RFT (*p* < 0.001, *r* = − 0.568), RF-CSA (*p* < 0.001, *r* = − 0.568), BBT (*p* < 0.001, *r* = − 0.539) and BB-CSA (*p* < 0.001, *r* = − 0.691) showed statistically significant differences between the two groups, as shown in Table 3.

**Table 3:** Differences and correlation analysis of ultrasound parameters in sarcopenia.

Variables	No-sarcopenia group (*n* = 30)	Sarcopenia group (*n* = 46)	Statistic	*P*	*r* (95%CI)
DTei (cm)	0.24 ± 0.05	0.19 ± 0.06	*t* = 3.441	<0.001	−0.406(−0.583,–0.192)
DTF (%)	18.19% (15.84, 22.88)	18.18% (11.74, 29.41)	*Z* = –0.149	0.882	0.018(−0.215,0.249)
DE (cm)	1.69 ± 0.56	1.37 ± 0.59	*t* = 2.412	0.018	−0.269(−0.472,–0.040)
ICMT (cm)	0.370 ± 0.097	0.33 ± 0.10	*t* = 1.770	0.081	−0.198(−0.411,0.035)
ICMTF (%)	11.96% (7.97, 17.15)	12.18% (5.72, 17.95)	*Z* = –0.122	0.903	−0.015(−0.246,0.218)
RAT (cm)	0.83 ± 0.18	0.77 ± 0.22	*t* = 1.358	0.179	−0.180(−0.396,0.054)
RFT (cm)	1.25 (0.99, 1.75)	0.67 (0.53, 0.88)	*Z* = –4.916	<0.001	−0.568(−0.707,–0.388)
RF-CSA (cm^2^)	2.90 (2.10, 3.74)	1.71 (1.26, 2.17)	*Z* = –4.915	<0.001	−0.568(−0.707,–0.387)
BBT (cm)	1.89 (1.50, 2.37)	1.36 (1.16, 1.62)	*Z* = –4.666	<0.001	−0.539(−0.686,–0.351)
BB-CSA (cm^2^)	6.53 (5.83, 7.67)	4.49 (3.37, 5.05)	*Z* = –5.978	<0.001	−0.691(−0.795,–0.547)

DTei, diaphragm thickness of end-inspiratory; DTF, diaphragm thickening fraction; DE, diaphragm excursion; ICMT, intercostal muscle thickness; ICMTF, intercostal muscle thickening fraction; RAT, rectus abdominis thickness; RFT, rectus femoris thickness; RF-CSA, rectus femoris cross-sectional area; BBT, biceps brachii thickness; BB-CSA, biceps brachii cross-sectional area.

SWE parameters were only available for a subset of COPD patients, as some patients were bedridden or had limited mobility, preventing measurement using a bedside ultrasound machine. Intergroup analysis using the rank-sum test and Spearman’s correlation analysis indicated that sarcopenia in COPD patients was associated with D-SWV (*p* < 0.001, *r* = − 0.523), ICM-SWV (*p* = 0.040, *r* = 0.273), RA-SWV (*p* < 0.001, *r* = 0.509) and BB-SWV (*p* = 0.046, *r* = 0.334), as detailed in Table 4.

**Table 4:** Differences and correlation analysis of shear wave elasticity parameters in sarcopenia.

Variables	No-sarcopenia group (n = 21)	Sarcopenia group (n = 21)	Statistic	*P*	*r* (95%CI)
D-SWV (m/s)	2.95 ± 0.33	2.46 ± 0.51	*t* = 3.701	<0.001	−0.523(−0.718,–0.251)
ICM-SWV (m/s)	2.81 ± 0.48	3.30 ± 0.92	*t* = −2.144	0.040	0.273(−0.043,0.539)
RA-SWV (m/s)	2.73 ± 0.57	3.51 ± 0.76	*t* = −3.765	<0.001	0.509(0.234,0.709)
RF-SWV (m/s)	3.16 ± 0.61	3.29 ± 0.70	*t* = −0.656	0.515	0.020(−0.294,0.330)
BB-SWV (m/s)	2.44 ± 0.36	2.76 ± 0.61	*t* = −2.079	0.046	0.334(0.024,0.585)

D-SMV, diaphragm shear wave velocity; ICM-SMV, intercostal muscle shear wave velocity; RA-SWV, rectus abdominis shear wave velocity; RF-SMV, rectus femoris shear wave velocity; BB-SMV, biceps brachii shear wave velocity.

### Muscle ultrasound determinants for diagnosing sarcopenia in COPD patients

Our further logistic regression analysis of 2D-US parameters revealed that ICMTF (*p* = 0.041, OR = 6.738), BBT (*p* = 0.094, OR = 6.231), DTF (*p* = 0.050, OR = 3.505), DE (*p* = 0.047, OR = 0.312), RF-CSA (*p* = 0.004, OR = 0.127) and BB-CSA (*p* = 0.009, OR = 0.009) were the independent influences factors for diagnosing sarcopenia in COPD patients, as detailed in Table 5.

**Table 5:** Univariate and multivariate logistic regression analysis of two-dimensional ultrasound parameters in sarcopenia.

Variables	Univariate regression		Multiple factor regression	
	*P*	OR (95%CI)	*P*	OR (95%CI)
Gender				
Female		1.000 (Reference)		
Male	0.638	1.269 (0.470 ~ 3.430)		
BODE grade				
Grade 1		1.000 (Reference)		
Grade 2	0.308	0.321 (0.036–2.846)		
Grade 3	0.318	2.786 (0.373–20.819)		
Grade 4	0.056	7.000 (0.952–51.448)	0.325	
BMI	0.444	0.833 (0.523–1.328)		
DTei	0.002	0.421 (0.241–0.735)	0.853	
DTF	0.485	1.191 (0.730–1.944)	0.050	3.505 (1.000–12.289)
DE	0.025	0.551 (0.327–0.928)	0.047	0.312 (0.099–0.984)
ICMTei	0.085	0.654 (0.403–1.060)	0.500	
ICMTF	0.543	1.162 (0.717–1.882)	0.041	6.738 (1.085–41.843)
RAT	0.179	0.722 (0.449–1.161)		
RFT	<0.001	0.160 (0.067–0.382)	0.437	
RF-CSA	<0.001	0.211 (0.103–0.431)	0.004	0.127 (0.031–0.513)
BBT	<0.001	0.207 (0.097–0.444)	0.094	6.231 (0.734–52.926)
BB-CSA	<0.001	0.062 (0.017–0.225)	0.009	0.009 (0.000–0.309)

Subsequently, we additionally included elasticity ultrasound parameters in the logistic regression analysis of patients undergoing elasticity imaging and found that RA-SWV (*p* = 0.015, OR = 19.171), BB-SWV (*p* = 0.046, OR = 4.837), RF-CSA (*p* = 0.094, OR = 0.263), DTei (*p* = 0.046, OR = 0.197) and ICM-SWV (*p* = 0.048, OR = 0.165) were the independent influences factors for diagnosing sarcopenia in COPD patients, as detailed in Table 6.

**Table 6:** Univariate and multivariate logistic regression analysis of two-dimensional ultrasound combined elasticity parameters in sarcopenia.

Variables	Univariate regression			Multiple factor regression
*P*	OR (95%CI)	*P*	OR (95%CI)	
Gender				
Female		1.000 (Reference)		
Male	0.179	2.615 (0.644–10.614)		
BODE grade				
Grade 1		1.000 (Reference)		
Grade 2	0.290	0.182 (0.008–4.263)		
Grade 3	0.512	2.500 (0.162–38.599)		
Grade 4	0.149	7.000 (0.497–98.601)		
BMI	0.455	0.790 (0.425–1.468)		
DTei	<0.001	0.142 (0.046–0.445)	0.046	0.197 (0.040–0.970)
DTF	0.840	1.065 (0.577–1.967)		
DE	0.190	0.634 (0.320–1.254)		
D-SWV	0.004	0.246 (0.094–0.644)	0.972	
ICMTF	0.635	1.165 (0.620–2.189)		
ICM-SWV	0.051	2.095 (0.997–4.400)	0.048	0.165 (0.028–0.986)
RAT	0.057	0.505 (0.250 ~ 1.020)	0.323	
RA-SWV	0.004	4.293 (1.580–11.665)	0.015	19.171 (1.763–208.429)
RFT	0.075	0.507 (0.240 ~ 1.072)	0.412	
RF-CSA	0.002	0.209 (0.078–0.561)	0.094	0.263 (0.055–1.252)
RF-SWV	0.506	1.235 (0.663 ~ 2.300)		
BBT	0.051	0.496 (0.245 ~ 1.003)	0.390	
BB-CSA	0.003	0.145 (0.040–0.524)	0.619	
BB-SWV	0.054	2.028 (0.989–4.160)	0.046	4.837 (1.027–22.768)

### Model robustness and validation

**Figure 3:**
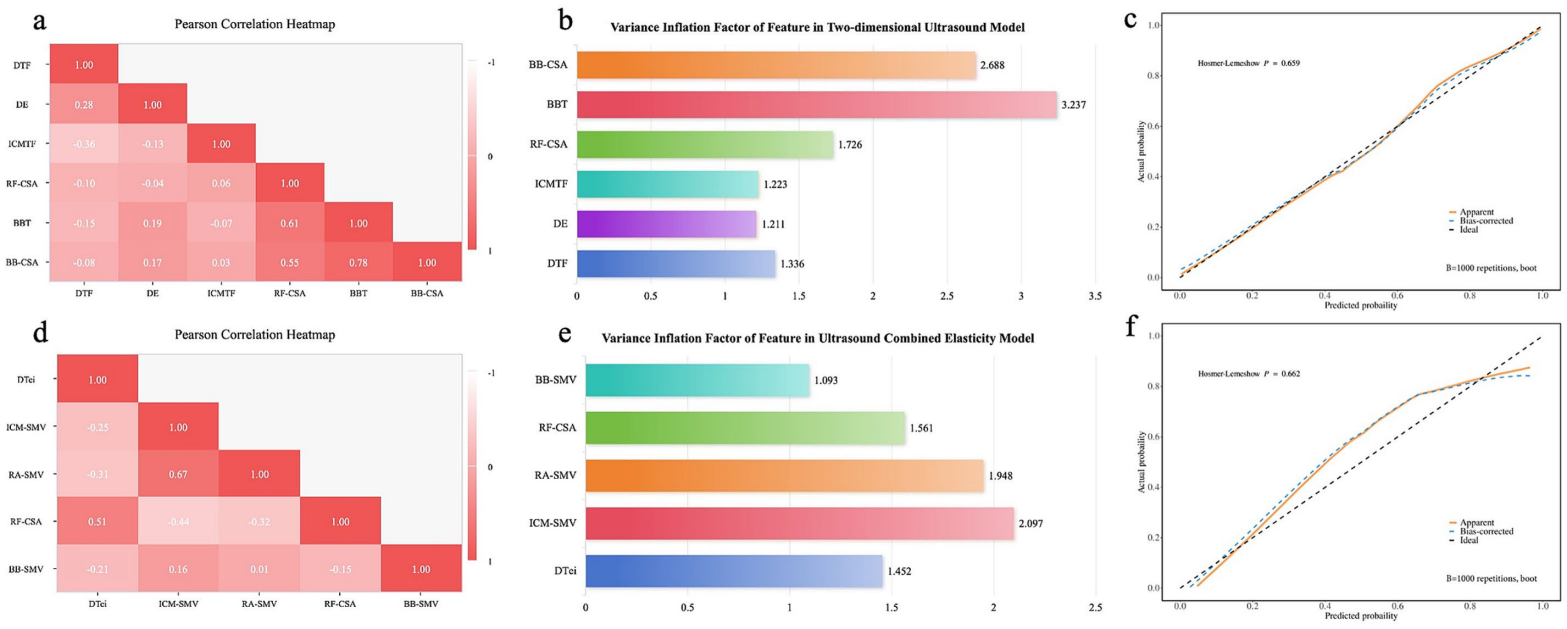
Assessment of multicollinearity and internal validation of models. This figure presents the evaluation of predictive models for both two-dimensional ultrasound model and combined elasticity model. **(a,d)** Pearson correlation heatmaps of the two-dimensional ultrasound **(a)** and combined elasticity **(d)** models, showing the relationships between model parameters. **(b,e)** Variance inflation factor (VIF) plots for the Two-dimensional ultrasound **(b)** and Combined elasticity **(e)** models. All VIF values are below 4, indicating no significant multicollinearity among the parameters. **(c,f)** Bootstrap-based Hosmer-Lemeshow test plots for the Two-dimensional ultrasound **(c)** and combined elasticity **(f)** models. All *p*-values are greater than 0.05, suggesting good model fit and robustness.

To assess the robustness and reliability of the predictive models, we performed a comprehensive analysis including multicollinearity analysis and internal validation. A combination of Pearson correlation heatmaps, variance inflation factor (VIF) plots, and bootstrap-based Hosmer-Lemeshow test results was used. The Pearson correlation heatmaps (Figures 3a,d) were generated to visualize the relationships between the parameters within the 2D-US model (Figure 3) and the combined elasticity model (Figure 3d), allowing us to assess the linear dependencies between features. The VIF plots (Figures 3b,e) were used to examine multicollinearity in both models, and all parameters demonstrated VIF values below 4, indicating minimal multicollinearity. Additionally, the bootstrap-based Hosmer-Lemeshow test (Figures 3c,f) was employed to evaluate the goodness-of-fit for the models, with *p*-values greater than 0.05 for both the 2D-US (Figure 3c) and combined elasticity (Figure 3f) models, suggesting good model fit. These analyses confirm the robustness of the predictive models, with minimal multicollinearity and reliable fit as demonstrated in the internal validation.

### Diagnostic performance of muscle ultrasound in diagnosing sarcopenia in COPD patients

**Figure 4:**
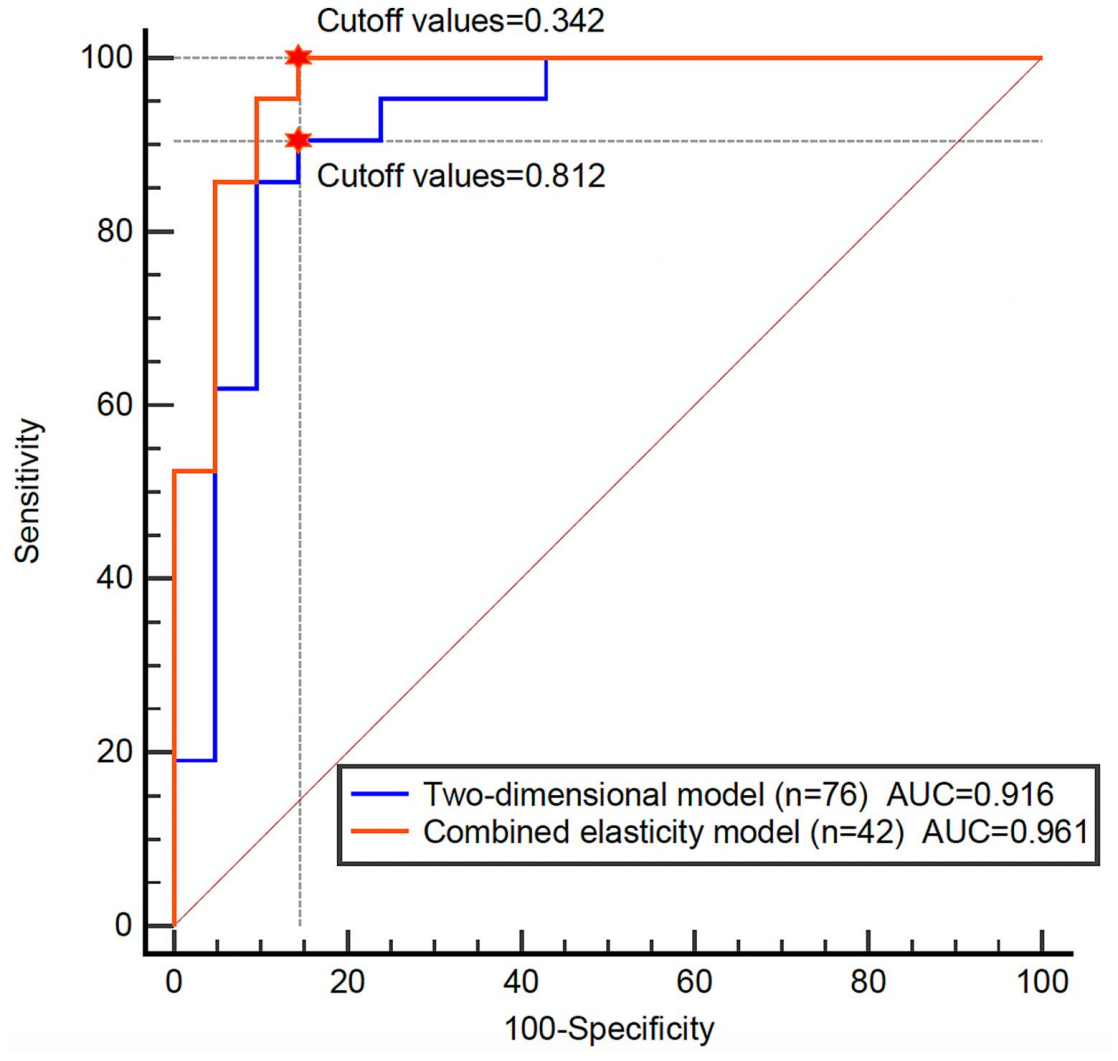
Comparison of AUC curves between two-dimensional ultrasound parameter model and combined elasticity model. The sample size and cutoff values are indicated in the figure. The red pentagram indicates the optimal cutoff point corresponding to the maximum Youden index.

To compare the diagnostic efficacy of 2D-US parameter models and combined with the SWE parameter models in identifying sarcopenia among COPD patients, we evaluated both models in a subset of 42 patients with complete data on both two-dimensional and SWE parameters (Figure 4).

The model based on logistic regression using 2D-US parameters alone yielded an area under the ROC curve (AUC) of 0.916 (95% CI: 0.857–1.000). The diagnostic performance included an accuracy of 0.857 (95% CI: 0.715–0.946), sensitivity of 0.905 (95% CI: 0.779–1.000), specificity of 0.810 (95% CI: 0.642–0.977), PPV of 0.826 (95% CI: 0.671–0.981), and NPV of 0.895 (95% CI: 0.757–1.000) (Table 7).

**Table 7:** Comparison of the diagnostic performance of the two models.

Indicators	Two-dimensional ultrasound	Ultrasound combined elasticity
AUC (95%CI)	0.916 (0.857–1.000)	0.961 (0.897–1.000)
Cutoff values	0.812	0.342
Accuracy (95%CI)	0.857 (0.715–0.946)	0.952 (0.838–0.994)
Sensitivity (95%CI)	0.905 (0.779–1.000)	0.905 (0.779–1.000)
Specificity (95%CI)	0.810 (0.642–0.977)	1.000 (1.000–1.000)
PPV (95%CI)	0.826 (0.671–0.981)	1.000 (1.000–1.000)
NPV (95%CI)	0.895 (0.757–1.000)	0.913 (0.798–1.000)

In comparison, the model incorporating both 2D-US parameters and elasticity parameters showed an AUC of 0.961 (95% CI: 0.897–1.000), with an accuracy of 0.952 (95% CI: 0.838–0.994), sensitivity of 0.905 (95% CI: 0.779–1.000), specificity of 1.000 (95% CI: 1.000–1.000), PPV of 1.000 (95% CI: 1.000–1.000), and NPV of 0.913 (95% CI: 0.798–1.000) (Table 7).

## Discussion

In this prospective study, we systematically evaluated the diagnostic value of 2D-US and SWE parameters of respiratory and superficial muscles in identifying sarcopenia among COPD patients. Our results demonstrate that both two-dimensional structural and elasticity-based ultrasound parameters provide valuable, noninvasive indicators for detecting sarcopenia, and that a combined model integrating these offers excellent diagnostic accuracy. These results highlight the potential of ultrasound technology as a promising tool for early identification of sarcopenia in COPD patients, which is essential for nutritional and therapeutic management.

Sarcopenia is increasingly recognized as a common and clinically significant complication of COPD (22). COPD-related sarcopenia is not only a matter of disuse but also a systemic expression of frailty and multimorbidity. It results from factors such as oxidative stress, systemic inflammation, microbiome dysbiosis, hypercapnia, hypoxia, and acidosis, which promote protein degradation and lead to widespread atrophy and dysfunction of respiratory and superficial muscles (23–25). These changes contribute to reduced exercise capacity, a higher risk of acute exacerbations, and elevated mortality. Sarcopenia in COPD is its strong association with malnutrition, which leads to reduced protein intake and anabolic resistance, further accelerating the decline in skeletal muscle mass and function (26).

Concurrently, sarcopenia and frailty are not only characterized by muscle loss and weakness but are also closely linked to impaired postural control, respiratory biomechanics, and the development of myofascial trigger points. Muscle weakness in both respiratory and postural muscles compromises postural stability, contributing to postural dysfunction that can worsen respiratory mechanics. Specifically, scapular dyskinesis and reduced spinal mobility may lead to inefficient ventilation and increased work of breathing. Furthermore, muscle dysfunction can result in the formation of myofascial trigger points, particularly in the respiratory and limb muscles, contributing to chronic pain and further limiting physical activity (27–30). These factors are critical in understanding the multifactorial nature of sarcopenia in COPD, highlighting the importance of a holistic approach to diagnosis and rehabilitation. Early detection of muscle dysfunction can help identify these issues and facilitate more targeted interventions that address both muscle strength and functional impairments, improving respiratory function and overall quality of life.

Traditional diagnostic tools for sarcopenia, such as DXA and BIA, are often limited in clinical practice due to concerns about cost, accessibility, and reduced accuracy in patients with fluid imbalance (15, 31–33). Although CT and MRI offer higher resolution and accuracy as diagnostic gold standards, both require specialized equipment, fixed examination locations and entail significant financial costs. This limits their widespread application in routine clinical practice, particularly in resource-constrained settings. In contrast, ultrasound offers a portable, noninvasive, and cost-effective alternative that is particularly suitable for patients with mobility limitations or respiratory compromise. Emerging evidence suggests that muscle ultrasound can be a practical tool not only for assessing muscle mass but also for monitoring the effectiveness of nutritional and rehabilitation interventions aimed at preserving muscle function in COPD patients.

The RF is one of the most commonly assessed muscles in ultrasound-based evaluations. Onishi et al. reported a strong correlation between RF-CSA and grip strength (34), while Fu et al., in a systematic review of 17 studies, found that RFT and RAT had moderate diagnostic accuracy for sarcopenia, as did RF-CSA and BB-CSA (15). Additionally, a reduction in DE has been linked to decreased skeletal muscle mass (35). Shinohara et al. reported that low skeletal muscle index (SMI) was negatively associated with DE and functional residual capacity (FRC) thickness during deep breathing (36). Wallbridge et al. also observed that intercostal muscle thickness measured by ultrasound moderately correlated with both FEV₁% predicted and RFT (18).

In our study, significant differences in mMRC scores, BODE scores, and FEV1% were observed between COPD patients with and without sarcopenia, further supporting the correlation between COPD and sarcopenia. And our findings demonstrate that several 2D-US parameters—including DTF, DE, ICMTF, BBT, RF-CSA, and BB-CSA—are independently associated with sarcopenia, and most of them show a negative correlation. These results are partly consistent with previous studies showing reduced respiratory and superficial muscle thickness and mobility in COPD patients and sarcopenia. Notably, ICMTF and DTF emerged as strong predictors, suggesting that respiratory muscle degradation may occur earlier or be more pronounced than superficial muscles loss in this population. BBT also showed significant predictive value, indicating its potential as a key superficial marker for sarcopenia assessment. Our results underscore the importance of assessing both respiratory and limb muscles in the diagnosis of sarcopenia. Given the integral role of respiratory muscles in COPD pathology, respiratory muscle assessment should be incorporated into routine sarcopenia screening, particularly in the context of malnutrition.

When SWE parameters were included into analysis, RA-SWV, BB-SWV, ICM-SWV, DTei, and RF-CSA emerged as independent predictors of sarcopenia in COPD patients. The combined model incorporating both SWE and structural ultrasound parameters demonstrated excellent diagnostic accuracy. These findings highlight the added value of assessing muscle stiffness using SWE, which provides complementary information beyond traditional morphological measurements. The significant predictive value of RA-SWV and BB-SWV suggests that changes in superficial muscle elasticity may reflect early alterations in muscle quality—such as fibrosis, impaired contractility, or shifts in muscle composition—that are not fully captured by thickness or cross-sectional area alone. With SWE technology, we can obtain more meaningful information from superficial muscle ultrasound, which is more reproducible than respiratory muscle ultrasound. This is particularly significant in the field of nutritional management, as elevated SWV values in muscles at rest typically indicate muscular rigidity. This condition frequently arises from fibrosis or fatty infiltration within muscle fibres. However, in voluntarily moving muscles such as the respiratory muscles, the SWV changes with the contraction of muscle fibers, reaching its peak value during maximum contraction. Consequently, a reduction in SWV typically indicates diminished muscle contractile function. Such alterations diminish the muscle’s capacity for effective contraction, leading to impaired muscular function and subsequently causing muscle weakness, atrophy and increases the risk of myofascial trigger points (18, 37–39). Ultrasound elastography can offer valuable insights into muscle health, guiding nutritional interventions aimed at improving muscle function and reducing muscle wasting in COPD patients.

Our findings suggest that ultrasound is a promising tool for assessing muscle structure and functional decline in COPD patients. The two-dimensional combined elasticity model showed excellent diagnostic performance, highlighting its potential for clinical use. Muscle stiffness, measured by SWE, provides complementary insights into muscle quality, crucial for early sarcopenia detection and intervention. From a nutritional perspective, understanding muscle stiffness can guide dietary strategies to address muscle fibrosis, such as protein and amino acid supplementation, microbiome-metabolome stratification, and personalized microbial modulation.

**Figure 5:**
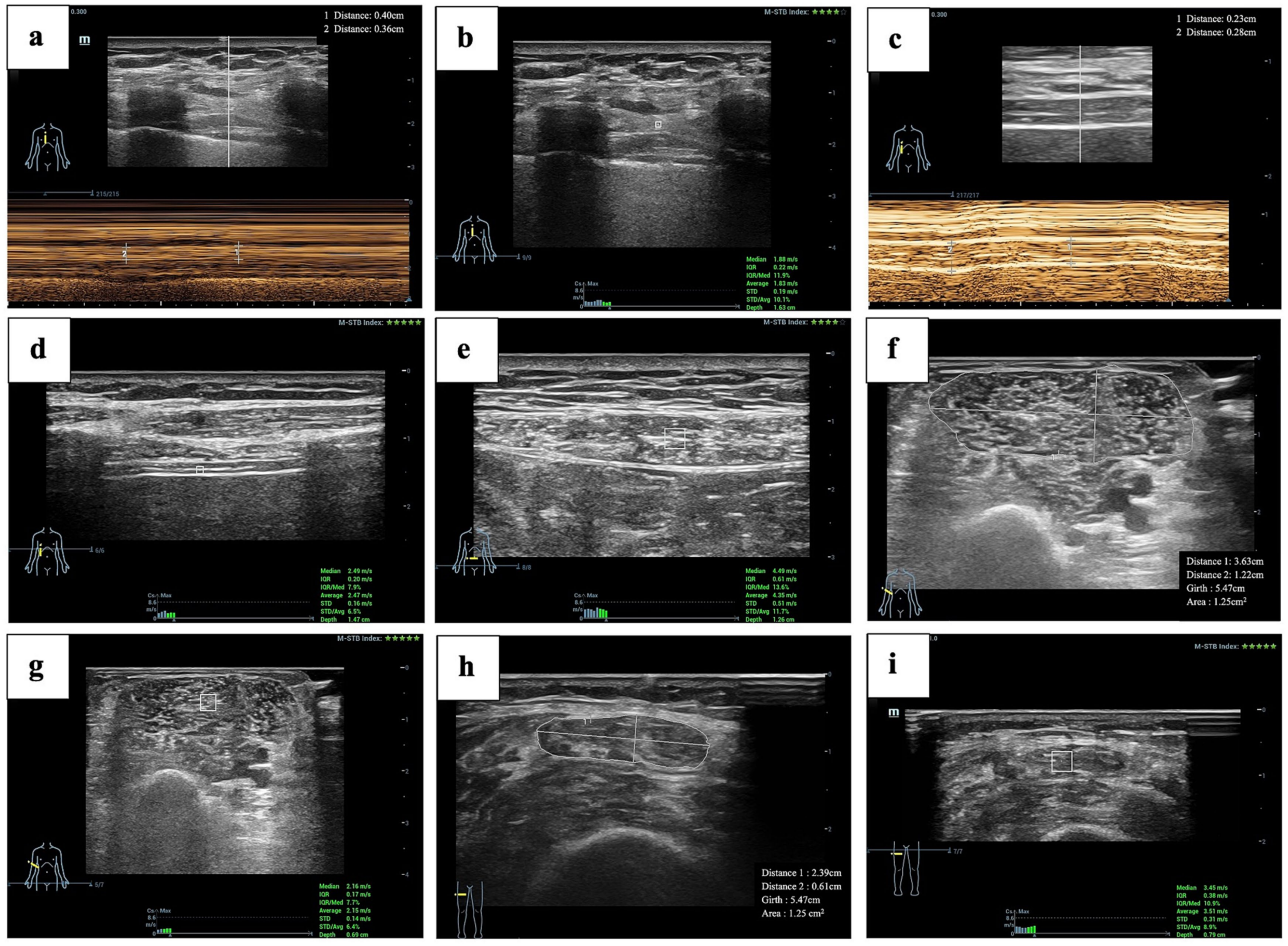
Clinical examples of multi-site multi-parameter ultrasound assessment. Clinical case of a 72-year-old male with COPD undergoing sarcopenia screening using multi-site and multi-parameter ultrasound. The patient has a history of over 30 years of COPD, a BMI of 19.03, calf circumference of 35 cm, and a right-hand dominant grip strength of 18.1 kg. Following ultrasound examination and clinical assessment, the patient was considered to have concomitant sarcopenia. Subsequently, a nutritionist devised a personalized intervention program. This program comprised a high-protein diet, probiotic supplements, and an exercise plan. **(a)** M-mode ultrasound measurement of intercostal muscle thickness at different phases of respiration. **(b)** Shear wave elastography (SWE) measurement of intercostal muscle shear wave velocity (SWV). **(c)** M-mode ultrasound measurement of diaphragm thickness at different phases of respiration. **(d)** SWE measurement of diaphragm shear wave velocity (D-SWV). **(e)** SWE measurement of rectus abdominis shear wave velocity (RA-SWV). **(f)** Cross-sectional area measurement of biceps brachii (BB-CSA). **(g)** SWE measurement of biceps brachii shear wave velocity (BB-SWV). **(h)** Cross-sectional area measurement of rectus femoris (RF-CSA). **(i)** SWE measurement of rectus femoris shear wave velocity (RF-SWV).

This study contributes to the growing body of evidence supporting ultrasound as a reliable, noninvasive tool for sarcopenia screening in COPD patients. Its portability and bedside applicability make it particularly valuable in settings with limited access to advanced imaging, like outpatient clinics and respiratory wards. A key strength of our study is its comprehensive approach, combining both functional (e.g., DTF, DE, DTei) and mechanical (e.g., SWV) assessments of respiratory muscles, an area often neglected in studies focused on limb muscles. This holistic evaluation emphasizes the importance of assessing muscle quality and function, not just mass, with significant nutritional implications (Figure 5). Integrating ultrasound into routine clinical practice could enable timely, targeted interventions, improving both quality of life and clinical outcomes for COPD patients.

**Figure 6:**
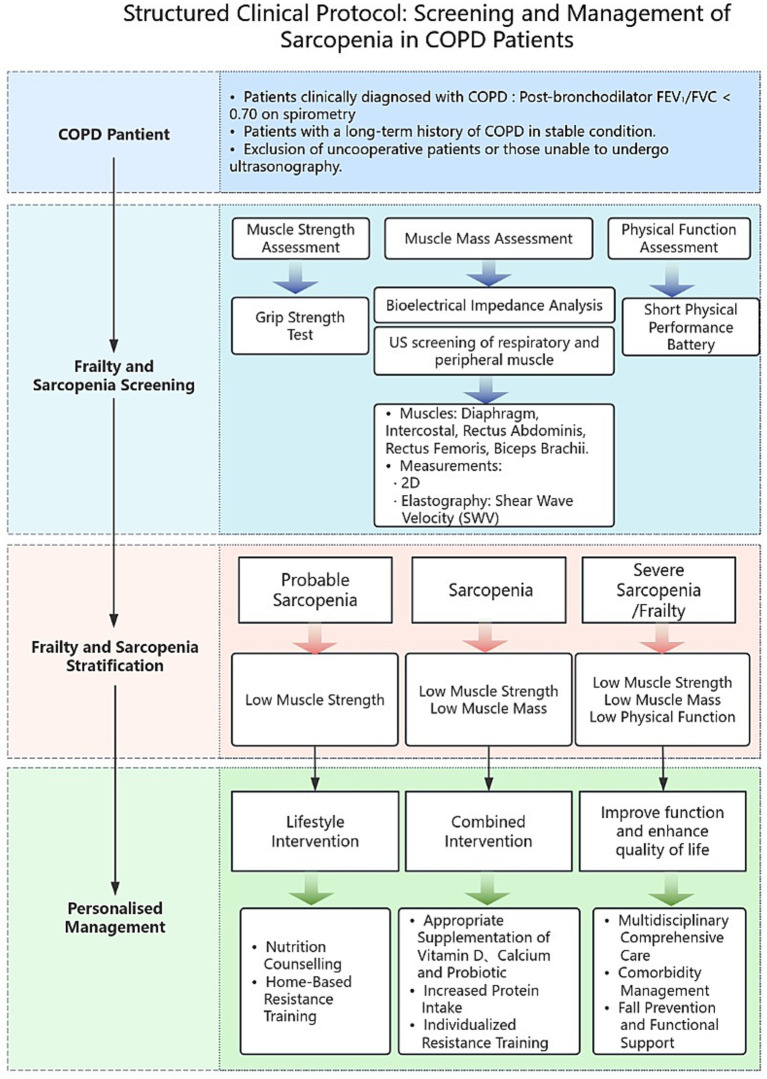
Sarcopenia screening and stratification workflow for COPD: from ultrasound to personalized care.

Early detection of sarcopenia via ultrasound can guide personalized nutritional interventions, such as optimizing protein intake, microbiome-metabolome stratification, individualized microbial modulation, initiating anabolic processes, and implementing supportive exercise therapy (Figure 6). These measures prevent further muscle loss and improve clinical outcomes, playing a vital role in nutritional prevention and rehabilitation for COPD patients (40–43).

While this study primarily focuses on ultrasound due to its practical, cost-effective, and bedside applicability, we also recognize the potential value of other imaging modalities such as CT and MRI. Specifically, CT imaging provides detailed insights into muscle and fat quality, including muscle attenuation and fat infiltration, which can contribute to a better understanding of muscle dysfunction in sarcopenia (44, 45). MRI, on the other hand, is particularly effective in evaluating muscle microstructure, including muscle fiber composition and intramuscular fat content. These imaging modalities are considered the gold standards in research for assessing muscle quality and microstructure, offering in-depth information on the pathophysiology of sarcopenia that ultrasound alone may not fully capture (46). Where conditions permit, combining multiple imaging techniques could provide a more comprehensive and detailed assessment of sarcopenia. This multimodal approach may offer complementary insights, improving diagnostic accuracy and allowing for a better understanding of the interplay between muscle mass, function, and quality in COPD patients.

Ultrasound undoubtedly holds significant promise for the assessment of sarcopenia in patients with COPD, offering a detailed investigation of various muscle groups. For example, functional ultrasound of the multifidus, scapular stabilizers, and shoulder girdle muscles can identify scapular movement disorders and postural muscle weakness, both of which are relevant to COPD rehabilitation (27). Additionally, ultrasound evaluation of the oral group muscles in the neck can aid in the functional assessment of sleep apnea syndrome in COPD patients (28). Moreover, ultrasound assessment of the lower limb and paraspinal muscles contributes to a multidimensional clinical evaluation of spinal movement and postural stability (29). This approach may also support the management of myofascial pain, trigger points, and physical therapy interventions, further enhancing the overall care of COPD and asthma patients (30, 47, 48).

Despite these promising results, our study has several limitations. First, it was conducted at a single center with a relatively small sample size, which may limit the generalizability of the findings and contribute to wide 95% confidence intervals for some variables in the multivariable logistic regression analysis. Larger, multicenter studies are needed for validation. Second, due to funding and clinical application limitations, the absence of SWE modules in bedside ultrasound devices resulted in missing SWE data for some bedridden patients, introducing potential selection bias. Further investigation is needed to assess the clinical utility of bedside SWE technology for more severe COPD cases. Third, while BIA was used to assess muscle mass due to practical constraints, it may be subject to error from fluid imbalances. Future studies should consider incorporating CT or MRI as gold standards for more accurate muscle mass measurement. Additionally, the lack of direct nutritional assessments, such as dietary intake or protein markers, is another limitation. Including these variables would provide a more comprehensive understanding of the relationship between nutrition and muscle health in COPD. Lastly, while ultrasound offers practical advantages, its accuracy can be influenced by operator experience and image acquisition variability, particularly for respiratory muscle ultrasound (49). Standardized protocols and longitudinal studies are needed to further validate ultrasound parameters in predicting clinical outcomes.

## Conclusion

In conclusion, this study demonstrates that ultrasound, when combined with SWE parameters, is a highly effective tool for detecting sarcopenia in COPD patients. The integration of structural and elastic ultrasound measures provides valuable insights into both muscle mass and quality, which are essential for comprehensive nutritional management. Early detection of sarcopenia, facilitated by ultrasound, can guide personalized interventions aimed at preserving muscle function and preventing further decline, ultimately improving clinical outcomes and quality of life for COPD patients.

## Data Availability

The raw data supporting the conclusions of this article will be made available by the authors, without undue reservation.

## Glossary

6-MWT: 6-min walk test
ASMI: Appendicular skeletal muscle index
BIA: Bioelectrical impedance analysis
BMI: Body mass index
BODE: Body-Mass Index, airflow Obstruction, Dyspnea, and Exercise capacity index
BB: Biceps brachii
COPD: Chronic obstructive pulmonary disease
CSA: Cross-sectional area
DE: Diaphragm excursion
DTei: Diaphragm thickness at end-inspiratory
DTee: Diaphragm thickness at end-expiratory
DTF: Diaphragm thickening fraction
DXA: Dual-energy X-ray absorptiometry
FEV1: Forced expiratory volume in 1 s
ICMTee: Intercostal muscle thickness at the end of expiration
ICMTei: Intercostal muscle thickness at the end of inspiration
mMRC: modified Medical Research Council
RA: Rectus abdominis
RF: Rectus femoris
SWE: Shear wave elastography
SWV: Shear wave velocity
